# The *Hypocrea jecorina (Trichoderma reesei) *hypercellulolytic mutant RUT C30 lacks a 85 kb (29 gene-encoding) region of the wild-type genome

**DOI:** 10.1186/1471-2164-9-327

**Published:** 2008-07-11

**Authors:** Verena Seidl, Christian Gamauf, Irina S Druzhinina, Bernhard Seiboth, Lukas Hartl, Christian P Kubicek

**Affiliations:** 1 Research Area Gene Technology and Applied Biochemistry, Institute of Chemical Engineering, Vienna University of Technology, Getreidemarkt 9/166-5, A-1060 Wien, Austria

## Abstract

**Background:**

The hypercellulolytic mutant *Hypocrea jecorina *(anamorph *Trichoderma reesei*) RUT C30 is the *H. jecorina *strain most frequently used for cellulase fermentations and has also often been employed for basic research on cellulase regulation. This strain has been reported to contain a truncated carbon catabolite repressor gene *cre1 *and is consequently carbon catabolite derepressed. To date this and an additional frame-shift mutation in the glycoprotein-processing β-glucosidase II encoding gene are the only known genetic differences in strain RUT C30.

**Results:**

In the present paper we show that *H. jecorina *RUT C30 lacks an 85 kb genomic fragment, and consequently misses additional 29 genes comprising transcription factors, enzymes of the primary metabolism and transport proteins. This loss is already present in the ancestor of RUT C30 – NG 14 – and seems to have occurred in a palindromic AT-rich repeat (PATRR) typically inducing chromosomal translocations, and is not linked to the *cre1 *locus. The mutation of the *cre1 *locus has specifically occurred in RUT C30. Some of the genes that are lacking in RUT C30 could be correlated with pronounced alterations in its phenotype, such as poor growth on α-linked oligo- and polyglucosides (loss of maltose permease), or disturbance of osmotic homeostasis.

**Conclusion:**

Our data place a general caveat on the use of *H. jecorina *RUT C30 for further basic research.

## Background

**Figure 1:**
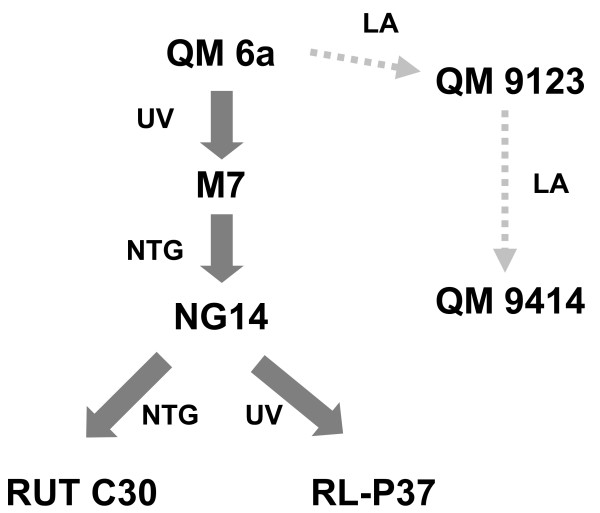
**Pedigree of strain *H. jecorina *RUT C30 and its relationship to the wild-type isolate QM6a.** Mutations into the strains were introduced by UV-light (UV), nitrosoguanidine (NTG) or linear accelerator (LA). The lineage leading to strain QM 9414 is also shown for comparison.

In modern biotechnology, many of the fermentations producing high volume/low price products make use of microbial strains which have been improved by classical mutagenesis using UV light or mutagenic chemicals. Information about the loci which became altered in the process of mutation and selection for improved product formation is scarce, if available at all. One notable exception is penicillin production by the fungus *Penicillium chrysogenum *[1-3], where the early mutation program has been shown to have removed detoxification reactions for the side chain precursor and has increased the biosynthetic capacity by amplification of the gene cluster for its production. In the case of the industrial cellulase producing fungus *Trichoderma reesei*, the anamorph of the pantropical ascomycete *Hypocrea jecorina*, all of the strains that are currently used on a commercial scale have been ultimately derived from one single isolate which was collected on the Solomon Islands during World War II [4,5]. The genetic basis of the respective mutations which led to enhanced cellulase production in these industrial strains is essentially unknown. However, B.S. Montenecourt and D.E. Eveleigh prepared two separate lines of mutants which led to the hypercellulolytic strains RUT C30 and RL-P37 ([5]; Fig. 1), of which *H. jecorina *RUT C30 has become the most frequently used strain for laboratory cellulase production [6-11]. In this strain two of its genetic changes have been described: one is a truncation in the *cre1 *gene encoding CRE1 the carbon catabolite repressor protein, which renders this strain carbon catabolite derepressed [12]; and another one leading to a frameshift mutation in the glycoprotein processing β-glucosidase II encoding gene [13]. Electrophoretic karyotyping showed that the two largest chromosomes in RUT C30 are somewhat smaller, whereas the other five chromosomes are somewhat larger, resulting in a total increase in genome size from 32.5 to 34.7 Mbps [14]. Gene mapping revealed a history of significant recombination events between the seven chromosomes, but no gene losses were observed so far [14,15]. The only exception that was noted was the absence of hybridization of one random clone (RC16) in RUT C30, which hybridized to chromosome IV in strain QM6a and chromosome I in strain QM9414 [14]. This suggests the presence of many more changes in RUT C30, which have not been uncovered until today.

Due to its carbon catabolite derepressed phenotype, *H. jecorina *RUT C30 has frequently been used as a reference strain in studies on the regulation of gene expression [16-18] or cell biology [19]. In a similar type of study, we have recently observed that the transcript of a gene was completely absent from RUT C30, and subsequently we found that also the gene was absent from this strain (Christian Gamauf, Christian P. Kubicek and Bernhard Seiboth, unpublished data). In the attempt to identify the reason for the absence of this gene, we discovered that *H. jecorina *RUT C30 lacks a large (85 kb) segment of genes present on scaffold 15 of the genomic sequence of the wild-type strain *H. jecorina *QM6a , [20]. The identification of these genes, and their correlation with changes in the phenotype of *H. jecorina *RUT C30 compared to strains QM6a and QM9414, are reported in this paper.

## Methods

### Fungal Strains and culture conditions

The *Hypocrea jecorina *strains QM6a (wild-type; ATCC 13631), QM9414 (early cellulase overproducing mutant; ATCC 26921), NG 14 (ATCC 56767) and RUT C30 (ATCC 56765) were used throughout this study. They were maintained on PDA slants (potato dextrose agar; Difco, Franklin Lakes, NJ, USA), and stock cultures kept at -80°C.

For shake flask cultures, 200 ml of Mandels Andreotti (MA) medium [21] with carbon sources added as given at the respective results sections, was suspended into 1 L Erlenmeyer flasks, inoculated with 5 × 10^7 ^spores, and incubated on a rotary shaker at 28°C and 200 rpm. To induce polyol dehydrogenases, glycerol 1% (w/v) was used as a carbon source. Cultures were harvested after 24 hrs by gentle filtration, and replaced onto fresh MA medium with either L-arabinose, erythritol or xylitol as an inducer (10 mM), and incubated for further 12 hrs. At this time they were harvested and used to prepare cell-free extracts (see below).

### Detection production of antimicrobial agents

Secretion of potential antimicrobial polyketides was tested by an agar diffusion method and plate confrontation tests. For the former, culture filtrates from various time points during growth- and stationary phase on D-glucose and lactose as a carbon source were sampled, proteins denatured by heating (100°C, 5 min), and the samples then concentrated to a tenth of their volume in a Speed Vac. They were then filtered through 20 μ filters (Millipore, Billerica, MA, USA) and pipetted into 8 mm holes punched into agar plates containing inocula of *Escherichia coli*, *Bacillus subtilis *and *Saccharomyces cerevisiae*. In the plate confrontation tests, 8 mm diameter agar plugs of mycelia of the two strains of *H. jecorina *were placed 5 cm apart from a respective colony of the same microbes. The presence of an antimicrobial component was indicated in both methods by a clearing zone.

### Nucleic acid isolation and hybridisation

Chromosomal DNA and total RNA were isolated as described [22,23]. Standard methods [24] were used for electrophoresis, blotting and hybridization of nucleic acids.

### PCR analysis

The open reading frames described in the results section were amplified from *H. jecorina *QM9414 and RUT C30 genomic DNA using the GoTaq^® ^system (Promega, Madison, WI, USA) with 0.4 μM of sequence specific primers (Table 1) and 2 mM MgCl_2_. The amplification protocol consisted of an initial denaturation step (2 min at 95°C) followed by 28 cycles of denaturation (1 min at 95°C), annealing (45 s at the primer specific temperature) and elongation (2–5 min at 72°) and was concluded by a final 7 min elongation step (72°C).

**Table 1:** PCR Primers used throughout this work

**Purpose**	**Target region**	**Primer name**	**Sequence (5' → 3')**
Determination of the are of deletion	ORF 1	rgx1startfw	TAAGTTTAGCTAAGGCAGAG
		rgx1startrv	AAATTAAAGAGGCTAGGCTG
	ORF 3	rgx1orf3fw	ACTCGTATGCTTGACTTTCTG
		rgx1orf3rv	CTATCTTGTTTAACCCAGTCAC
	ORF 4	rgx1orf4fw	CTCTTTACTCAATCGCCGAC
		rgx1orf4rv	CCAACAGCAGATTACGAGAC
	ORF 5	rgx1orf5fw	CTTATCCATTTCCGTGTTCC
		rgx1orf5rv	CTAGAATTCAAAGTCGCCAG
	ORF 10	rgx1orf10fw	TATAAGTCTGTTTGGTCCCTG
		rgx1orf10rv	GTATTACTCACGCTTTACCTG
	ORF 14	rgx1orf14fw	TAATAACCCAACCTCTACAC
		rgx1orf14rv	ACACGAGCAGAATATTAGTC
	ORF 15	rgx1orf15fw	GTACTCTAGAGACAGAATGGTGGCGCTATCGTC
		rgx1orf15rv	GTCAGGATCCAGAGCGGTATCAAGCAGTATCC
	ORF 16	rgx1orf16fw	ATGTCTACCTTACTGGATACTG
		rgx1orf16rv	CCGTCACATATTACAAGTTCTG
	ORF 20	rgx1orf20fw	ATCCACCTCATCGTTATTCC
		rgx1orf20rv	GTGGTTAAGAACAATGGAGC
	ORF 26	rgx1orf26fw	GTTGACACCATCTACTGCTG
		rgx1orf26rv	GCTTATCTACGCCGATTCTG
	ORF 28	rgx1orf28fw	GTGTTTAACCATAGCCAGAC
		rgx1orf28rv	TCTAGGTAAGCCTTCAAGAG
	ORF 29	rgx1orf29fw	GAACTCCCTAACTTCATCTCAG
		rgx1orf29rv	CAACCATCTCACTAGACCAC
	ORF 31	rgx1orf31fw	TTCTTGTCAACCCAACAGTC
		rgx1orf31rv	TTTCTACCACCTTTGAGCAG
	ORF 34	rgx1orf34fw	GATACGGTAGATATTCTTCC
		rgx1orf34rv	GAGAGTACATTCTAACTACC

Determination of the downstream end of the deletion	+500	orf31do05kFw	GAGGTACAGCGAATACAC
	+1000	orf31do1kFw	CAGATGGTGTTCAAGTTCTC
	+1500	orf31do15kFw	CTCTTGCTTCCATCAAATCAG
	+2000	orf31do2kFw	CGTCAAGTGTTATGTTGTCC
	+2500	orf31do25kFw	CGAGATGAAAGATTCACAGC
	+3000	orf31do3kFw	GAGGTATCGTGTTCAATGTC

Genome Walking		GWqm9414gsp1	CCTTATCACTACCTTCCACCTCCATCTTATACCC
		GWqm9414gsp2	CCTCCATCTTATACCCTCTACCCAATTCCC
		GWRUTC30gsp1	TACCGCCATCGCAGACTGTTCCCTTTC
		GWRUTC30gsp2	TCACTATGAGACGGCAG

*Cre1 *amplification		Cre1fw	TCTCTGGGCTCTCTTGTAACC
		CreIIr	TGCCACTCCTCCTCATGTCAT
		creF	GTACTTTGGCCCTCGCTGAG
		creR	CCAGACTGCATAAGGATTCCC
		creRUTr	AGCAATCAGGTGCAGATATCAC

### Genome Walking

To identify the 5' end of the deletion, the GenomeWalker™ Universal Kit (Clontech, Mountain View, CA, USA; [25]) was used. Briefly, this method first constructs pools of uncloned, adaptor-ligated genomic DNA fragments. Then, two PCR amplifications are preformed per library: the first uses the outer adaptor primer (AP1, provided by the manufacturer) provided in the kit and the outer, gene-specific primer (GWRUT C30gsp1; Table 1). The resulting PCR mixture is then used as a template for a secondary or "nested" PCR with the nested adaptor primer (AP2, provided by the manufacturer) and the nested gene-specific primer (GWRUT C30gsp2; Table 1). The DNA fragments were then cloned and sequenced. PCR amplifications were performed using the Long PCR Enzyme Mix (Fermentas, St.Leon-Rot, Germany). Distinct PCR products were amplified from libraries constructed with *Dra*I and *Stu*I endonucleases and sequenced (MWG Biotech, Ebersberg, Germany).

### Amplification and sequencing of the *cre1 *locus in *H. jecorina *RUT C30

The wild-type *H. jecorina cre1 *locus is located on scaffold 2, and its open reading frame (ORF) spans from 786955–789433 (ID 120117). Oligonucleotides used for the amplification of the *cre1.1 *mutation in strain RUT C30 and are given in Table 1.

### Enzyme extraction and assays

Preparation of cell free extracts and assay of xylitol and L-arabinitol dehydrogenases was performed essentially as described previously [26,27]. Erythritol dehydrogenase was measured in the same way as L-arabinitol dehydrogenase, but using 100 mM erythritol as a substrate.

### Microscopical analysis

Conidida from 7 – 10 day old cultures were collected and suspended in liquid Mandels Andreotti medium [21] containing either 1% or 10% (w/v) glucose and cultivated at 28°C. 50 μl drops of conidial suspension were placed on large cover slips and examined at room temperature by using differential interference contrast optics with a 60× (1.2 numerical aperture [NA]) water immersion plan apo objective on an inverted Nikon TE2000 microscope (Nikon, Kingston-Upon-Thames, UK). Images were captured with a Nikon DXM1200F digital camera and transferred into Adobe Photoshop software (version 10.0; Adobe Systems Inc., San Jose, CA, USA) for further processing.

### Biolog Phenotype Microarray analysis

Global carbon assimilation patterns were investigated using Biolog FF MicroPlate™ (Biolog Inc., Hayward, CA, USA), using the protocol published recently [28]. Briefly, *H. jecorina *strains were pregrown on 20 g·l^-1 ^malt extract agar, and 90 μl of a conidial suspension from them (75 ± 2% transmission at 590 nm) dispensed into each of the wells of a Biolog FF MicroPlate™ (Biolog Inc., Hayward, CA, USA). Inoculated microplates were incubated in the dark at 30°C, and percent absorbance determined after 12, 18, 24, 36, 42, 48, 66 and 72 h at 750 nm. Analyses were repeated at least three times for each strain.

### Statistical Analysis

Basic statistical methods such as multiple regression analysis and analysis of variance (ANOVA) as well as multivariate exploratory techniques (cluster and factor analyses) were performed using STATISTICA 6.1 (StatSoft, Inc., Tulsa, OK, USA) data analysis software system.

### Sequence analysis and phylogeny

The genome sequence of *H. jecorina *is available [29]. To screen the genome for genes missing in strain RUT C30, the "browse" function was used. Genes are identified by their protein ID number (search → gene models → protein id). Sequence analysis of the genes identified to be missing in *H. jecorina *RUT C30 was performed with InterProScan [30]) and SMART (/; [31]). Proteins with most similar sequences were identified by BLASTX[32]. For phylogenetic analysis, protein sequences were aligned using CLUSTALX 1.83 [33], the alignment edited with GENEDOC 2.6 [34] and the phylogenetic analysis performed in MEGA 3.1 [35].

## Results

### Identification of a genome fragment missing in *H. jecorina *RUT C30

**Figure 2:**
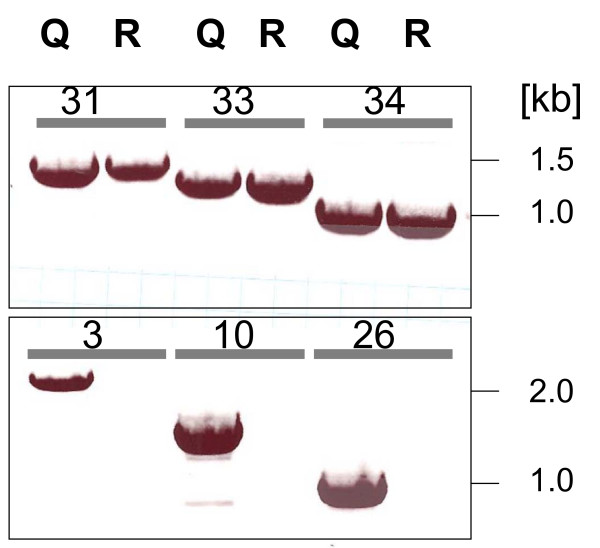
**Examples of the PCR strategy used to identify genes lacking in *H. jecorina *RUT C30 but being present in *H. jecorina *QM6a (genome sequence) and QM 9414 (experimental organism): Top row: genes present in both; bottom row: genes absent from RUT C30.** Genes amplified are indicated by the ORF no. as given in Tables 1 and 2. Q indicates strain QM 9414, R strain RUT C30.

The starting point of our analysis was a rhamnogalacturonase gene *rgx1 *(ID 122780) which is located on scaffold 15 in the *H. jecorina *genome database, and which is expressed in *H. jecorina *QM9414 but not in strain RUT C30 (C. Gamauf, C. P. Kubicek and B. Seiboth, unpublished data). Consequently, we tested by PCR whether this gene is actually present in the latter strain. Using the *rgx1*-specific primers given in Table 1, a clear product of expected size could be amplified from strain QM9414, but not from RUT C30 (Fig. 2a). Since this could indicate a gene deletion at this locus, we then designed primers for the amplification of the immediate 5' and 3' flanking genes (i.e. a monocarboxylate transporter, ID 109211; and an aldehyde dehydrogenase, ID 65142; respectively). As these two genes were apparently absent from strain RUT C30 too (Fig. 2b), a larger gene lesion was assumed. Therefore we screened for presence of genes with wider distance from the *rgx1 *locus until positive hits were found, and then reduced the intervals until the genes immediately flanking the gap could be identified. Thereby the first gene located 3' of the gap in RUT C30 was identified as a nitrilase, ID 64996. However, no gene could be found in the 5' direction of the gap, because even amplification of the most 5' located gene on scaffold 15 was not possible in strain RUT C30.

**Figure 3:**
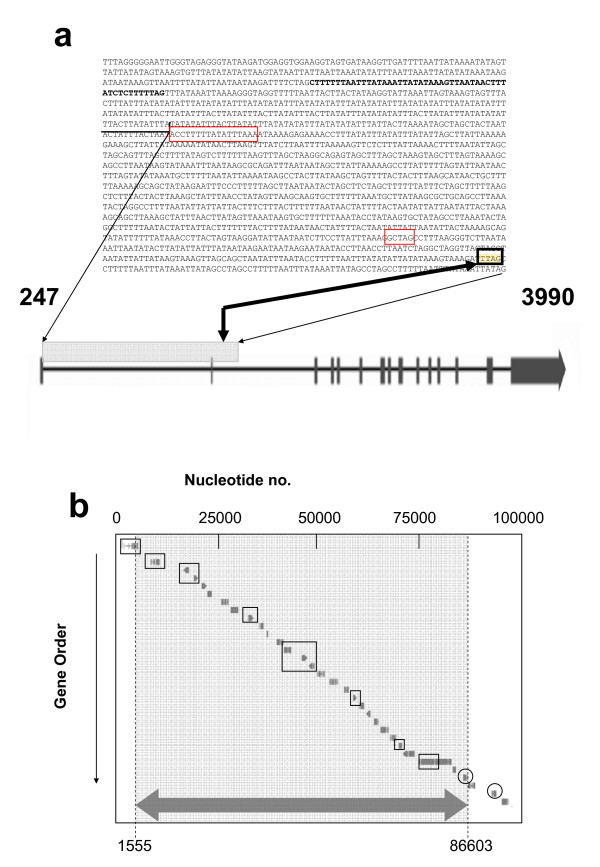
**Intron-exon organization of the first gene of scaffold_15, 15, and nt sequence of its first 1650 bp's.** The thin arrows show the location of the depicted nt sequence within the gene. The two short exons are boxed in red. The +1555 breakpoint is indicated by a thick arrow and boxed in black; (b) Scheme of the 5'-part of scaffold 15 of the genome of *H. jecorina*, and the area missing in strain RUT C30 (indicated by a grey background and the large arrow on the bottom). ORFs in square indicate those, whose absence has also been verified by PCR analysis, ORFs in circles indicate those which were verified to be present, respectively.

Since these data suggested that the gap may be continued on another, unknown scaffold, we applied a genome walking strategy. Primers were designed to hybridize within the intergenic region between the gene encoding a hypothetical protein, ID 79726, and the gene encoding a nitrilase, ID 64996 (primer GWRUT C30gsp1) and within the coding region of ID 79726 (primer GWRUT C30gsp2). Interestingly, in contrast to our assumptions, this method identified the 5' end of the gap close to the beginning of scaffold 15 in an AT-rich region (at +1555 bp; Fig. 3a). This breakpoint localizes within a large intron in the 5'-half of an ORF encoding a putative rhodanese-like protein ID 109199. This gene displays a high number of unusually long introns (Fig. 3a), and it is possible that these either represent annotation errors or it is a pseudogene. A closer investigation of this gene was beyond the scope of this paper, however.

The 3' end of the gap identified by genome walking corresponded to the region identified by PCR amplification and specified it at +86603 bp of scaffold 15 in the 5' nontranscribed area of the nitrilase-encoding gene (see above). Thus, this analysis provides evidence that *H. jecorina *RUT C30 contains an approximately 85 kb large gap on scaffold 15, which in *H. jecorina *QM6a [20] contains 29 ORFs (Fig. 3b) and that most of these genes are not present in the genome anymore.

### The 85-kb deletion is unlinked to the *cre1 *mutation

**Figure 4:**
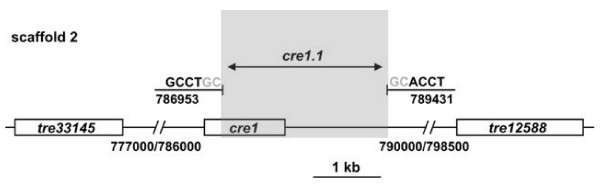
**A comparison of the *cre1 *locus in *Trichoderma reesei *QM6a and RUT C30.** The *cre1 *gene is located on scaffold 2. The respective location of the neighboring genes is also given. The *cre1.1 *mutation in RUT C30 comprises a region of 2478 bp, which is highlighted by a grey box. The two nucleotides given in grey could not be assigned unambiguously to one of the ends of the gap.

As the reason for this gene deletion in RUT C30 is unknown, we wondered whether it would be topologically related to the *cre1.1 *mutation. The *cre1 *locus in this strain has been shown to be truncated [11], but the exact length of the mutation and its genomic location has not yet been reported. A BLAST search of the *H. jecorina *genome sequence database with the cloned *cre1 *gene identified it to be located on scaffold 2: 786955–789433 (ID 120117), and thus distant from the locus of the lesion which was identified in this paper. In order to identify the *cre1.1 *mutation, we amplified and sequenced the *cre1 *locus in strain RUT C30. Using the primers Cre1fw and CreIIr (Table 1), PCR with QM9414 DNA resulted in a 3565 bp fragment as expected, whereas RUT C30 yielded a fragment of 1087 bp only. Sequencing of the fragment obtained with RUT C30, and its alignment with the sequence of scaffold 2 (Fig. 4) revealed the loss of a 2478 bp fragment which starts 3' of the region encoding the CRE1 zinc finger and reaches into a noncoding region. The coding region of the immediately following gene (tre12588) was not affected.

### *In silico *identification of the genes lacking in *H. jecorina *RUT C30

In order to evaluate the impact of the detected gene losses on the phenotype of *H. jecorina *RUT C30, we first performed a detailed *in silico *analysis of the encoded putative proteins (Table 2). Most of the ORFs could be aligned with proteins of known function, and only three of them encoded completely unknown proteins. Two genes encoded Cys_6_Zn(II)-type transcription factors, which are only found in fungi [36]. However, orthologues of these two transcription factors have not been described in any other fungus yet and their specific functions are therefore not known. The majority of the genes missing in *H. jecorina *RUT C30 encoded enzymes involved in primary metabolism (e.g. two aldehyde dehydrogenases, one aldo/keto reductase, one alcohol dehydrogenase, one glycerol dehydrogenase and one trehalase), and three transporters (a maltose permease, a monosaccharide transporter and an amino acid permease). The latter is very similar to a general amino acid permease that was characterized from *Amanita muscaria *[37]. Four other genes encoded extracellular enzymes (a glucan endo-1,6-β-glucosidase, a carbohydrate esterase, and the rhamnogalacturonase RGX1 that initially triggered this study). Finally, two of the genes missing in *H. jecorina *RUT C30 encoded proteins involved in cellular detoxification pathways, namely a multidrug efflux pump and a glutathione S transferase, and gene one encoded a class I reducing polyketide synthase.

**Table 2:** Identification of genes located on the 5' end of scaffold 15*

ORF	Location on scaffold	Protein ID	Putative function
1	247 – 3990	109199	Rhodanese-like protein
2	4265 – 4550	43418	Hypothetical protein
3	7135–9461	109201	FAD-linked oxidase
4	15618–16994	64959	Peptidase S26, signal peptidase
5	18217–19356	122778	Glycerol dehydrogenase GLD2
6	20262–21664	71817	Multidrug resistance protein
7	21775–22841	62215	carbohydrate esterase (family 4), imidase
8	25282–27143	65191	Maltose permease
9	27655–29638	109206	Heterokaryon incompatibility protein het-6
10	32246–33596	64906	Glucan endo-1,6-β-glucosidase (GH5)
11	35024–36114	49946	Glutathione S-transferase
12	36973–37308	65117	Ankyrin repeat protein
13	39475–41248	4726	Protein of the cytochrome P450 CYP2 family (phenylacetate-2 hydroxylase)
14	41375–43162	109211	Monocarboxylate transporter
15	45898–47405	122780	Rhamnogalacturonase RGX1
16	47630–49314	65142	Aldehyde dehydrogenase
17	49852–51907	64971	Aromatic and unpolar amino acid permease
18	52993–55245	71823	Cys6-transcription factor
19	56788–58016	6567	Aldo-keto reductase
20	59125–60081	65097	Alcohol dehydrogenase
21	60755–61994	79725	Cys6-transcription factor
22	62411–63580	65041	N2, N2-dimethylguanosine tRNA methyl transferase
23	64486–65668	64956	Aldehyde dehydrogenase
24	66149–68189	109219	Hypothetical protein, poorly conserved
25	68498–70244	65036	Cytochrome P450-dependent alkane hydroxylase
26	70809–71776	109221	Unknown protein, poorly conserved
27	71889–74905	25224	Acid trehalase GH65
28	76106–84410	65172	Polyketide synthase class 1, reducing
29	84689–85537	79726	Hypothetical protein, well conserved
30	87457–88602	64996	Nitrilase
31	88689–90473	122783	Cys6 transcription factor
32	94737–95939	65039	Sexual development inhibiting protein LsdA
33	97256–98976	65070	Cys6 transcription factor
34	101185–102595	65190	Nitrilase

* Genes 1–29 are are missing from *H. jecorina *RUT C30

### *H. jecorina *RUT C30 is impaired in the assimilation of α-glucans and -glucosides

**Figure 5:**
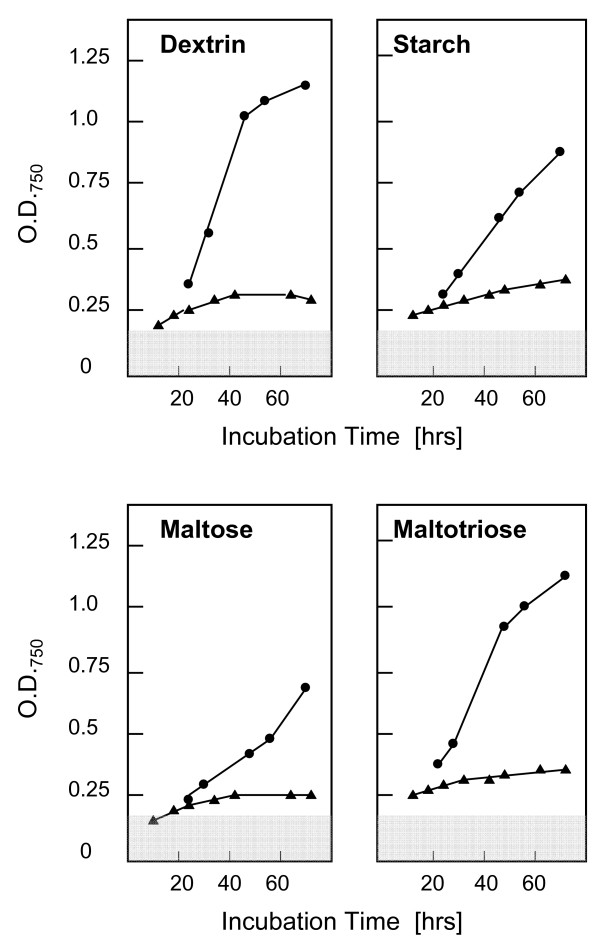
**Growth rates of *H. jecorina *strain QM6a (full circles) and strain RUT C30 (full triangles) on maltose, maltotriose, dextrin and starch, using the Biolog phenotype arrays system.** The grey area indicates the threshold of the water control. Data are shown are means of 3 separate experiments, which differed by less than ± 10 relative %.

The presence of a maltose permease in the missing genomic fragment raised the question whether this would have an impact of the growth of *H. jecorina *RUT C30 on α-linked glucans and glucosides. As can be seen from Fig. 5, growth on dextrin, starch, maltose and maltotriose was indeed strongly impaired in RUT C30, which is consistent with the absence of a maltose permease responsible for α-glucoside uptake. This interpretation is supported by the fact that *H. jecorina *– in contrast to several *Aspergillus *spp. – does not have multiple maltose permease genes (unpublished observations) and also lacks an extracellular α-glucosidase [38]. The present findings are therefore consistent with a metabolism of α-glucosides in *H. jecorina *by uptake and intracellular hydrolysis, which is impaired in RUT C30.

### *H. jecorina *RUT C30 displays several alterations in carbon source assimilation

**Figure 6:**
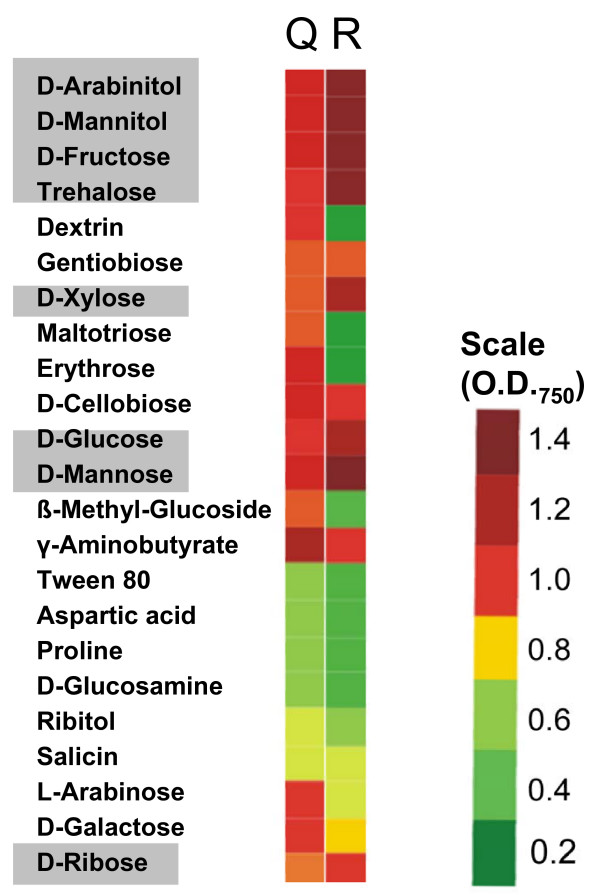
**Phenotype array analysis of carbon source profiles of *H. jecorina *QM6a (Q)and RUT C30 (R).** Only carbon sources where a difference to the parent strain QM6a was found are shown, and given in a color code. The OD_750 _refers to measurements at 48 hrs of growth, at which time the value is proportional to the growth rate (OD_750_/h) of the fungus on the respective carbon source. Carbon sources which are highlighted by a grey background are those which result in higher growth rates in RUT C30.

In view of the relatively strong abundance of metabolic genes in the genomic region which is missing in *H. jecorina *RUT C30, we performed a comprehensive analysis of its ability to assimilate (i.e. grow on) carbon sources using 95 carbon sources contained in the Biolog Phenotype Microarrays, and compared it to its wild-type strain QM6a. The data obtained (Fig. 6) identified several striking differences: strain RUT C30 had a strongly impaired growth on L-arabinose, L-erythritol, D-galactose and also 2-keto-D-gluconic acid. Interestingly, the opposite effect (= an enhancement of the assimilation rate) was also observed with some other carbon sources, e.g. glycerol, *N*-acetyl-β-D-glucosamine, D-mannitol, D-fructose, D-trehalose, D-mannose, D-ribose). This strongly reduced growth on L-arabinose, L-erythritol and also D-galactose suggested to us that one of the aldo/keto-reductases identified as lacking in strain RUT C30 (i.e. ID 65142, ID 6567, and ID 64956) could be involved in polyol assimilation. In order to test this hypothesis, we prepared cell free extracts from strains QM9414 and RUT C30, and tested these activities in cell-free extracts. As shown Table 3, both strains of *H. jecorina *had high NAD^+^-linked dehydrogenase activities with xylitol, L-arabinitol and erythritol and NADPH-linked dehydrogenase activities with D-xylose and L-arabinose as substrates, respectively. Activities with the other coenzyme (i.e. NADP with xylitol, L-arabinitol and erythritol; and NADH with D-xylose and L-arabinose) were negligible, with the exception of some NADP^+^-linked activity of strain RUT C30 on xylitol, which was absent from strain QM9414. In general, activities in strain RUT C30 were significantly higher. Only the NAD^+^-linked erythritol dehydrogenase activity was similar in both strains. These data indicate that the loss of the three aldo/ketoreductases in RUT C30 has apparently no effect on its metabolism of the major polyols and therefore cannot explain the different growth pattern of strain RUT C30 on L-arabinose and L-erythritol

**Table 3:** Polyol dehydrogenase activities in *H. jecorina *QM 9414 and RUT C30

	NAD		NADH		NADP		NADPH	
	QM9414	RUT C30	QM9414	RUT C30	QM9414	RUT C30	QM9414	RUT C30
D-Xylose			0.03 [± 0,025]	0.09 [± 0.01]			0.52 [± 0.03]	2.51 [± 0.04]
xylitol	1.1 [± 0.05]	2.4 [± 0.3]			< 0.005	0,14 [± 0.02]		
L-arabinose			0.018 [± 0.01]	0,021 [± 0.006]			0.3 [± 0.02]	1.15 [± 0.03]
L-arabinitol	0.45 [± 0.03]	0.75 [± 0.05]			< 0.005	0.05 [± 0.01]		
erythritol	0.23 [± 0.04]	0.25 [± 0.04]			< 0.005	< 0.005		

Components given in the first row were used as substrates, and the respective activities with NAD(P) or NAD(P)H are given in the rows under the respective coenzymes and strains. Open positions indicate that the experiment has not been done.

### *H. jecorina *RUT C30 favors high osmotic pressure

**Figure 7:**
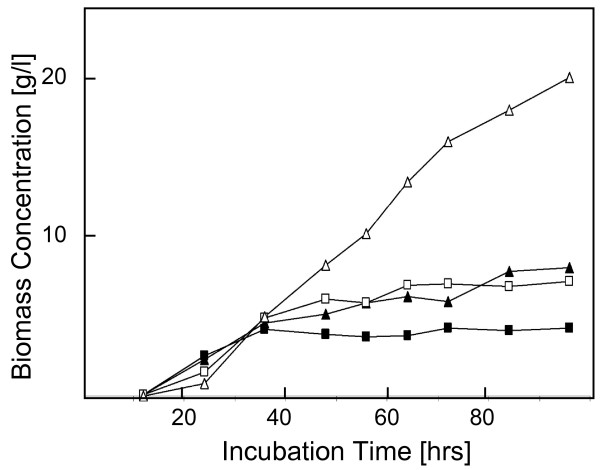
**Biomass formation of *H. jecorina *QM6a (full symbols) and RUT C30 (empty symbols) under osmotic stress evoked by 10% glucose.** (a) growth pattern in submerged culture on 1% (w/v) and 10% glucose (w/v) (squares and triangles, respectively). Values are from a single experiment, but data consistent with the claims made were obtained in at least two further experiments.

The glycerol dehydrogenase GLD2; EC 1.1.1.156; [39]), which is lacking in *H. jecorina *RUT C30, has been shown to be involved in glycerol formation during osmoadaptation in *A. nidulans *[40] and *H. atroviridis *([41]; there named GLD1). We were therefore interested to see whether the loss of glycerol dehydrogenase would render strain RUT C30 osmotically unstable. We grew strains QM9414 and RUT C30 in submerged culture on 1 and 10% (w/v) glucose. The results are shown in Fig. 7a: strain RUT C30 had a longer lag phase for growth but then accumulated about double the concentration of biomass than strain QM9414 under both conditions. The conversion yield Y_X/S _on 1% (w/v) glucose was 0.45 and 0.63 for strain QM9414 and RUT C30. While a yield over 0.5 can be explained by the utilization of amino acids in peptone for biomass formation [42], these data show that RUT C30 accumulates biomass more efficiently under both conditions. Yields on 10% (w/v) glucose were 0.08 and 0.22, respectively. This indicated that the loss of glycerol dehydrogenase did not render strain RUT C30 osmotically labile, but that it even performed better than strain QM9414 at high osmotic pressure.

### Spore volume increase during germination in *H. jecorina *RUT C30

**Figure 8:**
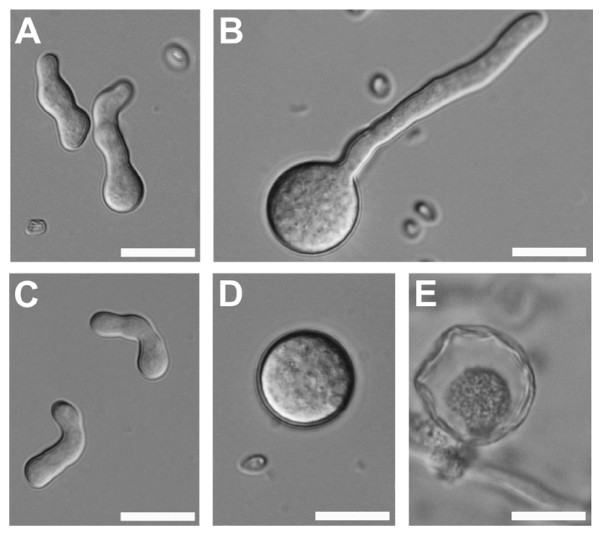
**DIC images of spore germination of *H. jecorina *grown in liquid medium with 1% glucose: (a) QM9414 and (b) RUT C30 and cultires grown under osmotic stress with 10% glucose: (c) QM9414 and (d) RUT C30.** (e) Swollwn RUT C30 spores that were unable to germinate underwent autophagic cell death. Bars = 20 μm.

In order to learn the reason for the prolonged lag phase in strain RUT C30, we microscopically examined the germination of its spores. This analysis revealed that RUT C30 spores first undergo considerable swelling and increase in size before they start to form a germ tube (Fig. 8). While spores of *H. jecorina *QM9414 showed a uniform spore diameter of 6 – 10 μm during spore germination, *H. jecorina *RUT C30 spores swelled up to a diameter of 20 – 30 μm, corresponding to an up to 50 – fold increase of spore volume (Fig. 8a–d). Interestingly, not all RUT C30 spores showed a swelling response and the extent of the swelling varied, resulting in a relatively homogenous distribution of spore diameters from ca. 10 to 25 μm. Germination was observed from swollen and not swollen spores and osmotic stress (10% carbon source) did delay germination but not influence the ratio of swollen to not swollen spores. However, although germination from even extremely swollen spores was observed, apparently not all swollen spores were able to enter the germination phase and during later growth stages a number of large spores that had undergone autophagic cell death [43] could be detected (Fig. 8e). The spore swelling and autophagy of swollen spores in *H. jecorina *RUT C30 could result in a delay of the formation of an interconnected mycelium and therefore explain the observed prolonged lag phase of RUT C30.

### *H. jecorina *RUT C30 lacks pigment formation

An intriguing observation during the cultivation experiments was that strain RUT C30 does not form the yellow pigment, which is characteristic for *H. jecorina *and other *Trichoderma *spp. from section *Longibrachiatum *[44]. This difference was observed both in late submerged cultures as well as during plate growth. We suspected that this could be due to the absence of the class I polyketide synthase ID 65172. In order to test this presumption, we subjected its amino acid sequence to phylogenetic analysis (NJ) with other polyketide synthases investigated by Kroken et al. [45]. In this analysis (data not shown), the *H. jecorina *polyketide synthase was determined to be member of clade I from the reducing polyketide synthases, thereby clustering most closely to *Bipolaris mayidis *PKS5, whose function is not known. Since none of the members of this cluster is known to be responsible for pigment formation, but some of them (e.g. the lovastatin synthase) synthesize antimicrobial polyketides, we also tested whether RUT C30 would be deficient in formation of an antimicrobial compound. However, using the agar diffusion assay and the confrontation assay, we could not detect any such compound in strain QM9414 and consequently also not in RUT C30 (data not shown). While the use of more sensitive methods such as MS may detect differences in secondary metabolite production between *H. jecorina *QM6a and RUT C30, our data show that the loss of this class I polyketide synthase does not influence the antimicrobial activity of *H. jecorina*.

### *H. jecorina *NG 14 has a full-size *cre1 *but lacks the 85 kb fragment

Both, *H. jecorina *RUT C30 and its ancestor NG 14, are mutants that underwent mutagenesis by nitrosoguanidin and were selected for growth on cellulose in the presence of glycerol (NG 14) and 2-desoxiglucose (RUT C30). We therefore wondered whether the loss of the 85 kb fragment and the truncation of *cre1 *were the result of one or both of these mutation steps.

**Figure 9:**
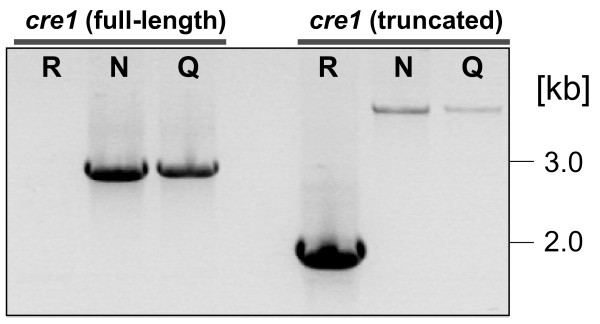
**Analysis of *cre1 *in *H. jecorina *RUT C30, NG 14 and QM9414 by PCR. Primers (given in table 1) used for '*cre1 *(full length)' were designed to form a PCR product (2.9 kb) only if the full length version of the gene is present but no PCR product for the truncated *cre1 *version detected in strain RUT C30.** For '*cre1 *(truncated)' the reverse primer was localized downstream of the *cre1 *gene, resulting in the formation of a 1.9 kb PCR product if a truncated *cre1 *gene was present in the genome and a 4.4 kb fragment if for full-length *cre1*. R indicates strain RUT C30, N strain NG 14 and Q strain QM9414.

In order to test the presence of the full-length or the truncated *cre1 *gene in NG 14, we designed primers creF and creR (table 1). CreR hybridizes in the 2.5 kb fragment of the *cre1 *locus that is absent in RUT C30 (see above) and should therefore result in amplification of a 2.9 kb fragment from the native *cre1 *gene only. Using these primers we could amplify the expected PCR product from QM9414 and NG 14 but not from RUT C30 (Fig. 9a). On the other hand using primer creF in combination with primer creRUTr, binding downstream of the *cre1 *truncation, we amplified a 1.9 kb fragment corresponding to the truncated *cre1.1 *gene from RUT C30, whereas the two other strains yielded a larger (4.4 kb) fragment corresponding to the native genomic locus (Fig. 9b). Consequently, we conclude that the *cre1 *truncation specifically occurred in *H. jecorina *RUT C30.

In order to test for the presence or absence of the 85-kb gene fragment, which is missing in RUT C30, the gene specific primers for ORFs 3, 4, 5, 10, 20 and 26 (table 1) were used. By means of these primers, we were unable to amplify a PCR product from strains NG 14 and RUT C30, whereas amplicons were obtained in the control with QM9414 (data not given), indicating that the large chromosome lesion is already present in the ancestor of RUT C30.

## Discussion

In the present work we have shown that the hypercellulolytic mutant *H. jecorina *RUT C30, in which only two mutations (in the carbon catabolite repressor protein CRE1 and the processing β-glucosidase II [12,13] had been described so far, carries a major deletion in its genome which comprises 85.048 bp including 29 open reading frames. Although this finding had not been detected so far, it is in accordance with earlier karyotyping results, which showed that the size and number of chromosomes in *H. jecorina *strain RUT C30 differed significantly from QM6a and QM9414 [14,15]. Unfortunately, none of the marker genes that were used in these studies was located on scaffold 15, and we were thus unable to identify the chromosome on which the 85 kb fragment described in this paper is lacking. However, despite of the fact that these 0.085 Mbp are a significant lesion, they are small compared to the changes in chromosome size determined by these authors. While the size determination in contour-clamped homogeneous electric field (CHEF) gel is not sensitive enough to distinguish between 0.1 and 0.2 Mbp, it is nonetheless possible that more deletions may be present in the genome of RUT C30.

The reason for this gene loss in RUT C30 is unclear. The genealogy leading to mutant RUT C30 involved three mutagenesis steps from the wild type strain (Fig. 1), of which the first one was simple UV mutagenesis whereas the subsequent two (from M7 to NG 14, and from NG 14 to RUT C30) involved mutagenesis by N-methyl-N'-nitro-N-nitrosoguanidine [5]. However, both agents usually do not lead to chromosome alterations or translocations. The results from genome walking have shown that the 5' site of the deletion is located in a region containing an about 1600 nt long palindromic AT-rich region (PATRR). PATRRs have been found to mediate genomic instability, thereby contributing to translocations, deletions and amplifications [46,47]. Carter et al. [15] have speculated that the lack of a sexual cycle and the need for mitotic pairing of chromosomes arising from there may increase the tolerance of mitosporic fungi to chromosome rearrangements. In the light of the above reasoning, PATRRs may be preferred regions for this. In *N. crassa*, this has been shown to be due to escape from het-c incompatibility [48]. The possibility that such rearrangements may regularly occur in *H. jecorina *would be consistent with similar data from other fungi [49], and also be consistent with results from the analysis of the genome of *H. jecorina *QM6a [20] which revealed a number of gene conversion events. In addition, such events could also occur during the regeneration of protoplasts after transformation with DNA (as shown for *Nectria haematococca *[50]), which would explain the high phenotypic diversity in *H. jecorina *transformants [27]. Our results with *H. jecorina *NG 14 show that the loss of the 85 kb gene fragment already occurred before the origin of RUT C30, and such an event must therefore have taken place in this or even an earlier mutant strain.

The structure of the gene encoding the rhodanese-like protein also supports such a scenario: this gene does not have any orthologues in fungi, but shows similarity to flavibacterial rhodanese-like proteins. It is conceivable that this gene arrived by horizontal gene transfer in an instable region, which led to the insertion to this unsual high number (14) of introns. The gene seems to be active, though, as the database lists 15 ESTs for it.

We were not able to predict the putative function of all genes which have been lost in RUT C30. Although we could therefore not to relate all of these genes to distinct phenotypic properties, for some of them clear correlations were obtained. One of them was the inability to grow on α-linked oligo- and polysaccharides, which we interpret to be due to the loss of the maltose permease gene (ID 65191). This finding implies that *H. jecorina *RUT C30 may not be a good source of enzyme production on carbon sources containing starch and other α-linked glycans, unless this deficiency is complemented by the corresponding gene from QM 9414.

Another intriguing finding during this study was that the loss of glycerol dehydrogenase GLD2 does not lead to an impaired osmotolerance. Consistent findings have been reported with a *gldB*-knock out strain of *A. nidulans *[40], where it was shown that this strain failed to accumulate glycerol during osmotic stress, but instead accumulated other polyols including D-mannitol, L-arabinitol and L-erythritol. It is therefore possible that other polyol dehydrogenase genes of *H. jecorina *can compensate for the loss of GLD2. However, the microscopic findings from this study, i.e. that RUT C30 displays a significant swelling of its conidia before it starts to germinate, indicate that osmotic homeostasis is impaired in this strain. A possible explanation for this finding would be that the compensating polyols (L-arabinitol, L-erythritol) are less fast metabolized, and thus lead to an increased osmotic pressure in the spores and delayed germination. The carbon source assimilation experiments also revealed that strain RUT C30 shows an enhanced growth rate on a number of simple carbon sources such as glycerol, *N*-acetylglucosamine, D-mannitol, D-fructose, D-trehalose, D-mannose, and D-ribose. Interestingly, there is evidence that some of them act as catabolite repressing carbon sources in *H. jecorina *(e.g. glycerol, [18]; fructose, [51]; mannose, unpublished data by L. Hartl, C.P. Kubicek and B. Seiboth). The phenotype in RUT C30 may be related to the loss of function of CRE1, and be due to the relief of catabolite repression by these carbon sources within their own catabolic pathways, most likely at the level of uptake. Sugar permeases of *H. jecorina *and other mitosporic fungi are known to be repressed by elevated levels of their substrates [52,53]. This property enables strain RUT C30 to grow faster at high sugar concentrations such as 6% lactose, a condition employed to make use of its superior cellulase forming capacity [54].

## Conclusion

In conclusion, we have identified a major genomic alteration in the hypercellulolytic mutant strain *H. jecorina *RUT C30, and have been able to correlate several of them with not yet apparent phenotypes of this strain. Likely, insights provided in this paper may only just be the beginning, and further such changes may be found when its genome would be subjected to a more thorough investigation. While the differences between the parent strain and RUT C30 do not interfere with the use of RUT C30 in biotechnology for the production of cellulases, it is clear that the use of this strain for basic research in physiology or molecular genetics is flawed. This is especially true for its use as a "carbon catabolite derepressed" mutant, because the truncation in its CRE1 protein clearly is only one of several more changes compared to its wild-type parent. Such a comparison may only be valid, if the results are compared to the mutant strain NG 14 in which the *cre1 *truncation has not yet occurred.

## Authors' contributions

VS performed the molecular work for this study, carried out the microscopy experiments and the investigation of strain NG 14, CG also performed molecular and physiological studies, and ISD performed and evaluated the Biolog phenotype analysis. LH and BS analyzed the *cre1 *locus. CPK supervised the project, wrote the draft of the manuscript and performed the final submission. All authors approved the final version of the manuscript.
